# Virtual Sensoring of Motion Using Pontryagin’s Treatment of Hamiltonian Systems

**DOI:** 10.3390/s21134603

**Published:** 2021-07-05

**Authors:** Timothy Sands

**Affiliations:** Sibley School of Mechanical and Aerospace Engineering, Cornell University, Ithaca, NY 14850, USA; tas297@cornell.edu

**Keywords:** virtual sensoring, physical sensors, smart/intelligent sensors, sensor technology and applications, sensing principles, signal processing in sensor systems

## Abstract

To aid the development of future unmanned naval vessels, this manuscript investigates algorithm options for combining physical (noisy) sensors and computational models to provide additional information about system states, inputs, and parameters emphasizing deterministic options rather than stochastic ones. The computational model is formulated using Pontryagin’s treatment of Hamiltonian systems resulting in optimal and near-optimal results dependent upon the algorithm option chosen. Feedback is proposed to re-initialize the initial values of a reformulated two-point boundary value problem rather than using state feedback to form errors that are corrected by tuned estimators. Four algorithm options are proposed with two optional branches, and all of these are compared to three manifestations of classical estimation methods including linear-quadratic optimal. Over ten-thousand simulations were run to evaluate each proposed method’s vulnerability to variations in plant parameters amidst typically noisy state and rate sensors. The proposed methods achieved 69–72% improved state estimation, 29–33% improved rate improvement, while simultaneously achieving mathematically minimal costs of utilization in guidance, navigation, and control decision criteria. The next stage of research is indicated throughout the manuscript: investigation of the proposed methods’ efficacy amidst unknown wave disturbances.

## 1. Introduction

**Figure 1:**
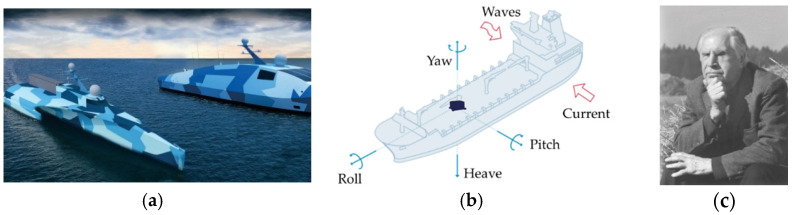
Representative motion measurement units for future ships depicted in (**a**) with measurement bases depicted in (**b**) are proposed to be augmented by virtual sensoring by minimization of Hamiltonian systems by the principles of Pontryagin depicted in (**c**). Future unmanned U.S. Navy vessels [3] Medium Unmanned Surface Vessel (MUSV) concept renderings in (**a**) from shipbuilder Austal USA. Photo Credit: Austal USA. Boat motion monitoring [4] uses measurement bases depicted in (**b**) whose graphic is from cited reference modified by author. Photo (**c**) of Lev Pontryagin from the archive of the Steklov Mathematical Institute [2] used with permission (30 June 2021).

Inertial measurement units provide continuous and accurate estimates of motion states in between sensor measurements. Future unmanned naval vessels depicted in Figure 1a require very accurate motion measurement units including active sensor systems and inertial algorithms when active sensor data is unavailable. State observers are duals of state controllers used for establishing decision criteria to declare accurate positions and rates and several instantiations are studied here when fused with noisy sensors, where theoretical analysis of the variance resulting from noise power is presented and validated in over ten-thousand Monte Carlo simulations.

The combination of physical sensors and computational models to provide additional information about system states, inputs, and/or parameters, is known as virtual sensoring. Virtual sensoring is becoming more and more popular in many sectors, such as the automotive, aeronautics, aerospatial, railway, machinery, robotics, and human biomechanics sectors. Challenges include the selection of the fusion algorithm and its parameters, the coupling or independence between the fusion algorithm and the multibody formulation, magnitudes to be estimated, the stability and accuracy of the adopted solution, optimization of the computational cost, real-time issues, and implementation on embedded hardware [1].

The proposed methods stem from Pontryagin’s treatment of Hamiltonian systems, rather than utilization of classical or modern optimal estimation and control concepts applied to future naval vessels as depicted in (Figure 1) [2,3,4].

Typical motion reference units conveniently have accuracies on the order of 0.05 (in meters and degree for translation and rotation, respectively, as depicted in Figure 1b for representative naval vessels as depicted in Figure 1a). These figures of merit are aspirational for the virtual sensor that must provide accurate estimates whether active measurements are available to augment the algorithm. Lacking active measurements, the algorithm is merely an inertial navigation unit, while with active measurements, the algorithm becomes an augmented virtual sensor. This manuscript investigates virtual sensoring by evaluating several options for algorithms, resulting estimated magnitudes, accuracy of each solution, optimization of resulting costs of motion, and sensitivity to variations like noise and parameter uncertainty of the translational and rotational motion models investigated (both simplified and high-fidelity). Algorithms are compared using various decision criteria to compare approaches for consideration of usage as motion reference units potentially aided by global navigation systems.

Noting the small size of motion measurement units, simple algorithms are preferred to minimize computational burdens that can increase unit size. Motion estimation and control algorithms to be augmented by sensor measurements are based on well-known mathematical models of translation and rotation from physics, both presented in equations. In 1834, the Royal Society of London published two celebrated papers by William R. Hamilton on Dynamics in the Philosophical Transactions. Ref. [5] The notions were slowly adopted, and not presented relative to other thoughts of the age for nearly seventy years [6], but quickly afterwards, the now-accepted axioms of translational and rotational motion were self-evidently accepted by the turn of the twentieth century [7,8,9,10] as ubiquitous concepts. Half a century later [11,12], standard university textbooks elaborate on the notions to the broad scientific community. Unfortunately, the notions arose in an environment already replete with notions of motion estimation and control based on classical proportional, rate, and integral feedback, so the fuller utilization of the first principals languished until exploitation by Russian mathematician Pontryagin [13]. Pontryagin proposed to utilize the first principles as the basis for treating motion estimation and control as the classical mathematical feedback notions were solidifying in the scientific community. Decades later, the first-principal utilization proposed by Pontryagin are currently rising in prominence as an improvement to classical methods [14]. After establishing performance benchmarks [15] for motion estimation and control of unmanned underwater vehicles, the burgeoning field of deterministic artificial intelligence [16,17] articulates the assertion of the first-principles as “self-awareness statements” with adaption [18,19] or optimal learning [20] used to achieved motion estimation and control commands. The key difference between the usage of first principals presented here follows. Classical methods impose the form of the estimation and control (typically negative feedback with gains) and they have very recently been applied to railway vehicles [21], biomechanical applications [22], and remotely operated undersea vehicles [23], electrical vehicles [24], and even residential heating energy consumption [25] and multiple access channel usage by wireless sensor networks [26]. Deterministic artificial intelligence uses first principals and optimization for all quantities but asserts a desired trajectory. Meanwhile the proposed methods in this manuscript leave the trajectory “free” and calculate an optimal state and rate trajectory for fusion with sensor data and calculates optimal decision criteria for estimation and controls in the same formulation.

This manuscript seeks to use the same notion, assertion of the first principals (via Pontryagin’s formulation of Hamiltonian systems) in the context of inertial motion estimation fused with sensor measurements (that are presumed to be noisy). Noise in sensors is a serious issue elaborated by Oliveiera et al. [27] for background noise of acoustic sensors and by Zhang et al. [28] for accuracy of pulse ranging measurement in underwater multi-path environments. Barker et al. [29] evaluated impacts on doppler radar measurements beneath moving ice. Thomas et al. [30] proposes a unified guidance and control framework for Autonomous Underwater Vehicles (AUVs) based on the task priority control approach, incorporating various behaviors such as path following, terrain following, obstacle avoidance, as well as homing and docking to stationary and moving stations. Zhao et al. [31] very recently pursued the presently ubiquitous pursuit of optimality via stochastic artificial intelligence using particle swarm optimization genetic algorithm, while Anderlini et al. [32] used real-time reinforcement learning. Sensing the ocean environment parallels the current emphasis in motion sensing, e.g., Davidson et al.’s [33] parametric resonance technique for wave sensing and Sirigu et al.’s [34] wave optimization via the stochastic genetic algorithm. Motion control similarly mimics the efforts of motion sensing and ocean environment sensing, e.g., Veremey’s [35] marine vessel tracking control, Volkova et al.’s [36] trajectory prediction using neural networks, and the new guidance algorithm for surface ship path following proposed by Zhang et al. [37]. Virtual sensory will be utilized in this manuscript where noisy state and rate sensors are combined to provide smooth, non-noisy, accurate estimates of state, rate, and acceleration, while no acceleration sensors were utilized. A quadratic cost was formulated for acceleration, since accelerations are directly tied to forces and torques and therefore fuels.

*“…condition of the physical world can either be ‘‘directly’’ observed (by a physical sensor) or indirectly derived by fusing data from one or more physical sensors, i.e., applying virtual sensors”*.

[38]

Thus, the broad context of the field is deeply immersed in a provenance of classical feedback driving a current emphasis on optimization by stochastic methods. Meanwhile this study will iterate options utilizing analytic optimization including evaluation of the impacts of variations and random noise in establishing the efficacy of each proposed approach. Analytical predictions are made of the impacts of applied noise power, and Monte Carlo analysis agrees with the analytical predictions. Developments presented in this manuscript follow the comparative prescription presented in [39], comparing many (eleven) optional approaches permitting the reader to discern their own preferred approach to fusion of sensor data with inertial motion estimation:

1. Validation of simple yet optimal inertial motion algorithms for both translation and rotation derived from Pontryagin’s treatment of Hamiltonian systems when fused with sensor data that is assumed to be noisy.
2. Validation of high-fidelity optimal (nonlinear, coupled) internal motion algorithms for rotation with translation asserted by logical extension derived from Pontryagin’s treatment of Hamiltonian systems when fused with sensor data that is assumed to be noisy;
3. Validation of three approaches for sensor data fused with the proposed motion estimation algorithm (not using classical feedback in a typical control topology): *pinv*, *backslash*, and *LU inverses* derived from Pontryagin’s treatment of Hamiltonian systems when fused with sensor data that is assumed to be noisy;
4. Comparison of each proposed fused implementation algorithm to three varieties of classical feedback motion architectures including linear-quadratic optimal tracking regulators, classical proportional plus velocity feedback tuned for performance specification and manually tuned proportional plus integral plus derivative feedback topologies, where these classical methods are utilized as benchmarks for performance comparisons when fused with sensor data that is assumed to be noisy.
5. Comparisons are made based on motion state and velocity errors, algorithm parameter estimation errors, and quadratic cost functions, which map to fuel used to create translational and rotational motion.
6. Vulnerability to variation is evaluated using ten-thousand Monte Carlo simulations varying state and rate sensor noise power and algorithm plant model variations, where noise power is tailored to the simulation discretization, permitting analytic prediction of the impacts of variations to be compared to the simulations provided.
7. Sinusoidal wave action is programmed in the same simulation code to permit future research, and inclusion of such is indicated throughout the manuscript.

Appendix A, Table A1 contains a consolidated list of variables and acronyms in the manuscript.

## 2. Materials and Methods

Inertial navigation algorithms use physics-based mathematics to make predictions of motion states (position, rate, acceleration, and sometimes jerk). The approach taken here is to utilize the mathematical relationships from physics in a feedforward sense to produce optimal, nonlinear estimates of states that when compared to noisy sensor measurements yield corrected real-time optimal, smooth, and accurate estimates of state, rate, and acceleration. Sensors are modeled as ideal with added Gaussian noise and the smooth estimates will be seen to exhibit none of the noise. The optimization of the estimates will be derived using Pontryagin’s optimization.

**Figure 2:**
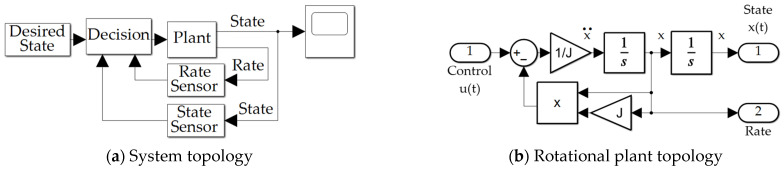
SIMULINK simulation program topologies used to generate the results in Section 3: (**a**) Overall system topology used to simultaneously produce state and rate estimates integrated with noisy sensors and additionally optimal control calculations; (**b**) Euler’s moment from Equation (1) elaborated in [5,6,7,8,9,10,11,12] describing rotational motion (notice the nonlinear coupled motion).

Motion control algorithms to be augmented by sensor measurements are based on well-known mathematical models of translation and rotation from physics, both presented in Equation (1), where both high-fidelity motion models are often simplified to identical double-integrator models where nonlinear coupling cross-products of motion are simplified, linearized, or omitted by assumption. The topologies are provided in Figure 2. Centrifugal acceleration is represented in Equation (1) by $-m\omega \times \left(\omega \times r^{\prime }\right)$. Coriolis acceleration is represented in Equation (1) by $-2m\omega \times v^{\prime }$. Euler acceleration is represented in Equation (1) by $m\dot{\omega }\times r^{\prime }$. In this section, double-integrator models are optimized by Pontryagin’s treatment of Hamiltonian systems, where the complete (not simplified, linearized, or omitted) nonlinear cross-products of motion are accounted for using feedback decoupling. Efficacy of feedback decoupling of the full equations of motion is validated by disengaging this feature in a single simulation run to reveal the deleterious effects of the coupled motion when not counteracted by the decoupling approach.

(1) $$\tau =I\dot{\omega }+\underset{\begin{array}{c} \;rotation\;due\;\\ to\;rotating\;\\ reference \end{array}}{\underbrace{\omega \times I\omega }}\;↔\;F=ma^{\prime }+\underset{translation\;due\;to\;rotating\;reference}{\underbrace{m\dot{\omega }\times r^{\prime }-2m\omega \times v^{\prime }-m\omega \times \left(\omega \times r^{\prime }\right)}}$$

where

F, τ external force and torque, respectively
m, I mass and mass moment of inertia, respectively
ω,$\;\dot{\omega }\;$angular velocity and acceleration, respectively
$r^{\prime }$,$\;v^{\prime },\;a\prime \;$position and velocity, and acceleration relative to rotating reference
$\tau =I\dot{\omega }\;$and F=m a′ are double integrator plants
ω×Iω cross-product rotational motion due to rotating reference frame
$m\dot{\omega }\times r^{\prime }\;$cross-product translation motion due to rotating reference frame
$-2m\omega \times v^{\prime }\;$cross-product translation motion due to rotating reference frame
$-m\omega \times \left(\omega \times r^{\prime }\right)\;$cross-product translation motion due to rotating reference frame.

### 2.1. Problem Scaling and Balancing

Consider problems whose solution must simultaneously perform mathematical operations on very large numbers and very small numbers. Such problems are referred to as poorly conditioned. Scaling and balancing problems are one potential mitigation where equations may be transformed to operate with similarly ordered numbers by scaling the variables to nominally reside between zero and unity. Scaling problems by common, well-known values permits single developments to be broadly applied to a wide range of state spaces not initially intended. Consider problems simultaneously involving very large and very small values of time ($\bar{t}$), mass ($\bar{m}$)/mass moments of inertia ($\bar{I}$), and/or length ($\bar{r}$). Normalizing by a known value permit variable transformation such that newly defined variables are of similar order, e.g., $t\equiv \frac{\bar{t}}{t_{f}},\;I\equiv \frac{\bar{I}}{I_{system}}=J\equiv \frac{\bar{J}}{J_{system}},m\equiv \frac{\bar{m}}{m_{system}},r\equiv \frac{\bar{r}}{r^{\prime }}$ where r indicates generic displacement units like x,y, or angle. Such scaling permits problem solution with a transformed variable mass and inertia of unity value, initial time of zero and final time of unity, and state and rate variables that range from zero to unity making the developments here broadly applicable to any system of particular parameterization.

### 2.2. Scaled Problem Formulation

The problem is formulated in terms of standard form described in Equations (2)–(8), where x(·),v(·) are the decision variables. The endpoint cost $E\left(x\left(t_{f}\right)\right)$ is also referred to as the Mayer cost. The running cost F(x(t),u(t)) is also referred to as the Lagrange cost (usually with the integral). The standard cost function J[x(·), u(·)] is also referred to as the Bolza cost as the sum of the Mayer cost and Lagrange cost. Endpoint constraints $e\left(x\left(t_{f}\right)\right)$ are equations that are selected to be zero when the endpoint is unity.

(2) $$x^{T}=\left[x,v\right],\;\;\;\;u=\left[u\right]$$

(3) $$\mathrm{Minimize}\;\;\;\;\;\;J\left[x\left(\cdot \right),\;v\left(\cdot \right),\;u\left(\cdot \right)\right]=E\left(x\left(t_{f}\right)\right)+\int_{0}^{t_{f}}F\left(x\left(t\right),u\left(t\right)\right)dt=\frac{1}{2}\int_{0}^{t_{f}}u^{2}dt$$

(4) $$\mathrm{Subject}\;\mathrm{to}\;\;\;\;{\dot{x}}_{1}=f_{1}\left(x\left(t\right),u\left(t\right)\right)=v$$

(5) $${\dot{x}}_{2}=f_{2}\left(x\left(t\right),u\left(t\right)\right)=\dot{v}=u$$

(6) $$\left(x_{0},v_{0}\right)=\left(0,0\right)$$

(7) $$\left(x_{f-1},v_{f},t_{f-1}\right)=\left(0,0,0\right)$$

(8) $$e\left(x\left(t_{f}\right)\right)=0$$

where

J[x(·), u(·)] cost function
$x^{T}=\left[x,v\right]\;$state vector of motion state x and rate v with initial condition
$x^{T}=\left[x,v\right]\;$$\left(x_{0},v_{0}\right)$ and final conditions $\left(x_{f-1},v_{f},t_{f-1}\right)=\left(0,0,0\right)$
u=[u] decision vector
H Hamiltonian operator corresponding to system total energy
$\lambda^{T}\;$adjoint operators, also called co-states (corresponding to each state)
$\upsilon^{T}\;$endpoint costates
$e\left(x\left(t_{f}\right)\right)\;$endpoint constraints.

### 2.3. Hamiltonian System: Minimization

The Hamiltonian in Equation (8) is a function of the state, co-state, and decision criteria (or control) and allows linkage of the running costs F(x,u) with a linear measure of the behavior of the system dynamics f(x,u). Equation (9) articulates the Hamiltonian of the problem formulation described in Equations (2)–(5). Minimizing the Hamiltonian with respect to the decision criteria vector per Equation (10) leads to conditions that must be true if the cost function is minimized while simultaneously satisfying the constraining dynamics. Equation (11) reveals the optimal decision u will be known if the rate adjoint can be discerned.

(9) $$H=F\left(x,u\right)+\lambda^{T}f\left(x,u\right)$$

(10) $$H=\frac{1}{2}u^{2}+\lambda_{x}v+\lambda_{v}u$$

(11) $$\frac{\partial H}{\partial u}=0\to u+\lambda_{v}=0$$

### 2.4. Hamiltonian System: Adjoint Gradient Equations

The change of the Hamiltonian with respect to the adjoint λ maps to the time-evolution of the corresponding state in accordance with Equations (12) and (13).

(12) $${\dot{\lambda }}_{x}=-\frac{\partial H}{\partial x}=0\to \lambda_{x}\left(t\right)=a$$

(13) $${\dot{\lambda }}_{v}=-\frac{\partial H}{\partial v}=\lambda_{x}\to {\dot{\lambda }}_{v}=\lambda_{x}\left(t\right)=a\to \lambda_{v}\left(t\right)=-at-b$$

The rate adjoint was discovered to reveal the optimal decision criteria, and the adjoint equations reveal the rate adjoint is time-parameterized with two unknown constants still to be sought. Together, Equations (11)–(13) form a system of differential equations to be solved with boundary conditions (often referred to as a two-point boundary value problem in mathematics).

### 2.5. Terminal Transversality of the Enpoint Lagrangian

The endpoint Lagrangian $\bar{E}$ in Equation (14) adjoins the endpoint function endpoint cost $E\left(x\left(t_{f}\right)\right)$ and the endpoint constraints functions $e\left(x\left(t_{f}\right)\right)$ in Equation (8) and provides a linear measure for endpoint conditions in Equation (7). The endpoint Lagrangian $\bar{E}$ exists at the terminal (final) time alone. The transversality condition in Equation (15) specifies the adjoint at the final time is perpendicular to the cost at the end point. In this problem, the endpoint cost $E\left(x\left(t_{f}\right)\right)=0$. These Equations (16) and (17) are often useful when seeking a sufficient number of equations to solve the system.

(14) $$\bar{E}=E+\upsilon^{T}e=\upsilon^{T}e=\upsilon_{x}\left(x_{f}-1\right)+\upsilon_{v}\left(v_{f}-0\right)=\upsilon_{x}\left(x_{f}-1\right)+\upsilon_{v}v_{f}$$

(15) $$\frac{\partial \bar{E}}{\partial x_{f}}=\lambda_{x}\left(t_{f}\right)$$

(16) $$\frac{\partial \bar{E}}{\partial x_{f}}=\lambda_{x}\left(t_{f}\right)=\upsilon_{x}$$

(17) $$\frac{\partial \bar{E}}{\partial v_{f}}=\lambda_{v}\left(t_{f}\right)=\upsilon_{v}$$

### 2.6. New Two-Point Boundary Value Problem

For the two-state system, four equations are required with four known conditions to evaluate the equations. In this instance, two Equations (3)–(10) have been formulated for state dynamics, two more Equations (18) and (19) have been formulated for the adjoints, and two more Equations (20) and (21) have been formulated for the adjoint endpoint conditions. Four known conditions, Equations (22)–(25) have also been formulated. Combining Equations (11) and (13) produce Equation (26).

(18) $$\dot{x}=v$$

(19) $$\dot{v}=u$$

(20) $${\dot{\lambda }}_{x}=0$$

(21) $${\dot{\lambda }}_{v}=-\lambda_{x}$$

(22) x(0)=0

(23) v(0)=0

(24) x(1)=1

(25) v(1)=0

Evaluating Equation (27) with Equation (23) produces the value c=0. Evaluating Equation (28) with Equation (22) produces the value d=0. Evaluating Equation (27) with Equation (25) produces Equation (29), while evaluating Equation (28) with Equation (24) produces Equation (30).

(26) $$\dot{v}=-\lambda_{v}\left(t\right)=at+b$$

(27) $$v=\int \dot{v}dt=\frac{1}{2}at^{2}+bt+c$$

(28) $$x=\int vdt=\frac{1}{6}at^{3}+\frac{1}{2}bt^{2}+ct+d$$

(29) $$v\left(1\right)=\frac{1}{2}a+b=0$$

(30) $$x\left(1\right)=\frac{1}{6}a+\frac{1}{2}b=1$$

Solving the system of two Equations (29) and (30) produces a=−12 and b=6. Substituting Equation (26) into Equation (11) with a and b produces Equation (31), and substitution of a and b into Equations (27) and (28), respectively, produce Equations (32) and (33) the solution of the trajectory optimization problem.

(31) $$u^{*}\left(t\right)=-12t+6$$

(32) $$v^{*}\left(t\right)=-3t^{2}+6t$$

(33) $$x^{*}\left(t\right)=-2t^{3}+3t^{2}$$

Equations (31)–(33) constitute the optimal solution for quiescent initial conditions and the state final conditions (zero velocity and unity scaled position). To implement a *form of feedback* (not classical feedback), consider leaving the initial conditions non-specific in variable-form as described next. 

### 2.7. Real-Time Feedback Update of Boundary Value Problem Optimum Solutions

Classical methods utilize feedback of asserted form u=−Kx for state variable x, where the decision criteria (for control or state estimation/observer) and gains K are solved to achieve some stated performance criteria. Such methods are used in Section 3 and their results are established as benchmarks for comparison. So-called modern methods utilize optimization problem formulation to eliminate classical gain tuning substituting optimal gain selection but retaining the asserted form of the decision criteria. Such methods are often referred to as “linear-quadratic optimal” estimators or controllers. These estimators are also presented as benchmarks for comparison, where the optimization problem equally weights state errors and estimation accuracy.

**Figure 3:**
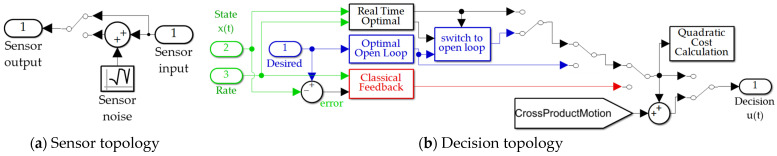
Simulink systems for noisy sensors and decision criteria (guidance or control) subsystem: (**a**) noisy sensor subsystem; (**b**) decision topology (guidance or control).

Alternative use of feedback is proposed here (whose simulation is depicted in Figure 3b). Rather than classical feedback topologies asserting u=−Kx utilization of state feedback in formulating the estimator or control’s decision criteria, this section proposes re-labeling the current state feedback as the new initial conditions of the two-point boundary-value problems used to solve for optimal state estimates or control decision criteria in Equations (22) and (23). The solution of (26)–(28) using the initial values of (22) and (23) manifest in values of the integration constants: a=−12 and b=6. As done in real-time optimal control, the values of the integration constants are left “free” in variable form, and their values are newly established for each discrete instance of state feedback (re-labeled as new initial conditions). This notion is proposed in Proposition 1, whose proof expresses the form of the online calculated integration constants that solve the new optimization problem. The two constants $\hat{a}$ and $\hat{b}$ are utilized in the same decision Equation (31) where the estimates replace the formerly solved values of the boundary value problem resulting in Equation (40).

**Proposition** **1.**  *Feedback may be utilized not in closed form to solve the constrained optimization problem in real time.*

(34) $$x=\frac{1}{6}at^{3}+\frac{1}{2}bt^{2}$$

(35) $$v=\frac{1}{2}at^{2}+bt$$

(36) $$x_{f}=\frac{1}{6}at_{f}^{3}+\frac{1}{2}bt_{f}^{2}$$

(37) $$v_{f}=\frac{1}{2}at_{f}^{2}+bt_{f}$$

**Proof** **of** **Proposition** **1.**  Implementing Equations (34)–(37) in matrix form as revealed in Equation (38) permits solution for the unknown constants as a function of time as displayed in Equation (39), and subsequent use of the unknown constants form the new optimal solution from the current position and velocity per Equation (40).

(38) [t036t022t01t022t01016121112110]⏟T{abcd}⏟p={x0v010}⏟q

(39) {a^b^c^d^}=[t036t022t01t022t01016121112110]−1{x0v010}

(40) $$u^{*}\equiv \hat{a}t+\hat{b}$$

**Figure 4:**
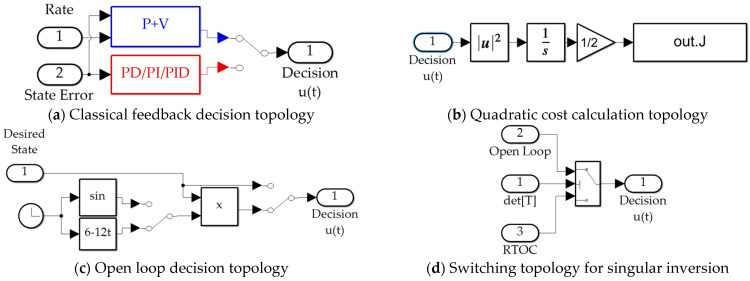
SIMULINK subsystems: (**a**) implementation of classical feedback methods; (**b**) calculation of quadratic cost; (**c**) open loop decision topologies (sinusoidal and optimal); (**d**) switching function to disengage use of real-time parameter updates when the matrix in Equation (39) is rank deficient. Notice in subfigure (**c**) sinusoidal wave input is coded using the identical time-index of the rest of the simulation. The next stages of future research will utilize this identical simulation to investigate efficacy of the proposed virtual sensoring amidst unknown wave actions.

In Section 3, estimation of $\hat{a}$ and $\hat{b}$ becomes singular due to the inversion in Equation (39) as approaching the terminal endpoint, where switching to Equations (31)–(33) is implemented as depicted in Figure 4d to avoid the deleterious effects of singularity when applying Proposition 1. The cases with switching at singular conditions are suffixes with “*with switching*” in the respective label.

### 2.8. Feedback Decoupling of Nonlinear, Coupled Motion Due to Cross Products

The real-time feedback update of boundary value problem optimum solutions is often used in the field of real-time optimal control, but a key unaddressed complication remains the nonlinear, coupling cross-products of motion due to rotating reference frames. Here, a feedback decoupling scheme is introduced, allowing the full nonlinear problem to be addressed by the identical scaled problem solution presented, and such is done without simplification, linearization, or reduction by assumption. In proposition 2, feedback decoupling is proposed to augment the optimal solution already derived. The resulting modified decision criteria in Equation (42) is utilized in simulations presented in Section 3 of this manuscript, but a single case omitting Proposition 2 is presented to highlight the efficacy of the approach.

**Proposition** **2.**  *The real-time optimal guidance estimation and/or control solution may be extended from the double-integrator to the nonlinear, coupled kinetics by feedback decoupling as implemented in Equation (41).*

(41) $$\tau =I\dot{\omega }+\underset{\begin{array}{c} \;rotation\;due\;\\ to\;rotating\;\\ reference \end{array}}{\underbrace{\omega \times I\omega }}$$

**Proof** **of** **Proposition** **2.**  For nonlinear dynamics of translation or rotation as defined in Equation (1), where the double-integrator is augmented by cross-coupled motion due to rotating reference frames, the same augmentation may be added to the decision criteria in Equation (40) using feedback of the current motion states in accordance with Equation (42). The claim is numerically validated with simulations of “cross-product decoupling” that are nearly indistinguishable from open loop optimal solution, and a single case “without cross-product decoupling” is provided for comparison.

(42) $$u^{*}\equiv \hat{a}t+\hat{a}+\omega \times I\omega$$

### 2.9. Analytical Prediction of Impacts of Variations

Assuming Euler discretization (used in the validating simulations) for output *y*, index *i* and integration solver timestep *h* Equation (43) would seem to indicate a linear noise output relationship. Equation (44) indicates the relationship for quiescent initial conditions indicating the results of a style draw. In a Monte Carlo sense (to be simulated) of a very large number *n*, Equation (45) indicates expectations from theory Equation (46) in simulation for scaled noise entry to the simulation to correctly reflect the noise power of the noisy sensors in the discretized computer simulation. Equation (46) was used to properly enter the sensor noise in the simulation (Figure 2a and Figure 3a).

(43) $$\dot{y}\left(t\right)=\frac{y_{i+1}-y_{i}}{h}=n_{i}\to y_{i+1}=y_{i}+hn_{i}$$

(44) $$y_{1}=\underset{0}{\underbrace{y_{0}}}+hn_{0}$$

(45) $$\frac{1}{N}\overset{N}{\underset{i=1}{\sum }}y_{1i}^{2}=\sigma_{y}^{2}\to \frac{1}{N}\overset{N}{\underset{i=1}{\sum }}{\left(hn_{o,i}\right)}^{2}=h^{2}\frac{1}{N}\overset{N}{\underset{i=1}{\sum }}n_{o,i}^{2}\to \sigma_{y}^{2}=h^{2}\sigma_{n}^{2}$$

(46) $$\mathrm{let}\;\sigma_{sim}^{2}=\frac{\sigma_{n}^{2}}{h}\to \sigma_{y}^{2}=h^{2}\sigma_{sim}^{2}=h^{2}\frac{\sigma_{n}^{2}}{h}=h\sigma_{n}^{2}\to \sigma_{sim}^{2}=\frac{\sigma_{n}^{2}}{h}$$

Assuming this implementation of noise power for a given Euler (ode1) discretization in SIMULINK, 1 − σ error ellipse may be calculated as Equation (47) for the system in canonical form in accordance with [40] and was implemented in Figure 3a and depicted on “scatter plots” in Section 3’s presentation of results of over ten-thousand Monte Carlo simulations.

(47) $$\sigma_{n_{state}}=\sqrt{\frac{\omega_{n}^{2}+4\zeta^{2}}{4\zeta \omega_{n}}}\;\sigma_{n_{rate}}=\sqrt{\frac{\omega_{n}^{3}+4\zeta^{2}\omega_{n}}{4\zeta }}$$

### 2.10. Numerical Simulation in MATLAB/SIMULINK

Validating simulations were performed in MATLAB/SIMULINK Release R2021a with Euler integration solver (ode1) and a fixed time step of 0.01 s, whose results are presented in Section 3, while this subsection displays the SIMULINK models permitting the reader to duplicate the results presented here. Sensor noise was added per Section 2.8. The classical feedback subsystem is displayed in Figure 4a. The optimal open loop subsystem implements Equation (31), and is elaborated in Figure 4b,c. The real time optimal subsystem implements Equations (42) and (31) augmented by feedback decoupling as in Equation (42). The “switch to open loop” subsystem switches when the matrix inverted in Equation (39) is singular indicated by a zero valued determinant and is elaborated in Figure 4d. The quadratic cost calculation computes Equation (3) and is elaborated in Figure 4b, while the cross-product motion feedback implements the cross product of Equation (42). The P + V subsystem and PD/PI/PID subsystems depicted in Figure 4a implement classical methods not re-derived here, but whose computer code is presented in Appendix B, Algorithms A1 and A2. 

**Figure 5:**
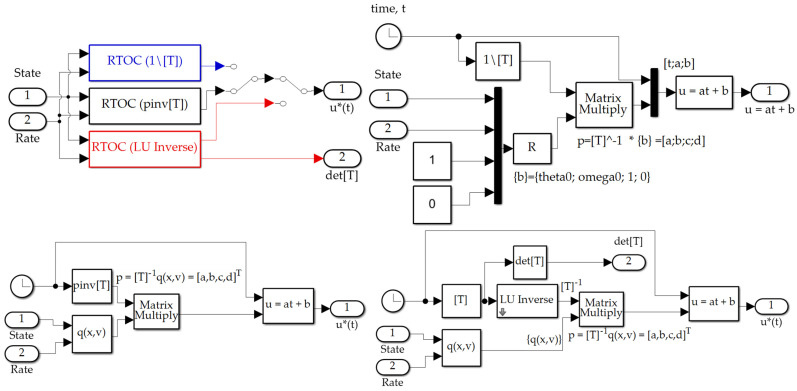
SIMULINK subsystems; (**a**) real-time optimal decision switching topology; (**b**) real-time optimal calculation of Equation (39) using *backslash\inverse*; (**c**) real-time optimal calculation of Equation (39) using *pinv inverse*; (**d**) real-time optimal calculation of Equation (39) using *LU inverse*.

Figure 5 displays the SIMULINK subsystems used to implement the three instantiations of real-time optimization (labeled RTOC from provenance in optimal control) where the switching displayed in Figure 3b permits identical simulation experiments to be performed with all conditions fixed, varying only the proposed implementation. The subsystems execute Equation (39) with three variations of matrix inversion: (1) MATLAB’s backslash “\”, (2) Moore-Penrose pseudoinverse (*pinv*), (3) *LU-inverse*.

Section 2.10 presented SIMULINK subsystems used to implement the equations derived in the section. Table 1 displays the software configuration used to simulate the equations leading to the results presented immediately afterwards in Section 3. 

## 3. Results

Section 2 derived several options for estimating state, rate, and control simultaneously as outputs of Pontryagin’s treatment of the problem formulated as Hamiltonian systems. Section 2.9 described implementation of sensor noise narratively, while Figure 3 illustrated the topological elaboration using SIMULINK including state and rate sensor with added Gaussian noise whose noise power was set in accordance with Section 2.9. SIMULINK subsystems were presented to aid repeatability (with callback codes in the Appendix B). Those subsystems were used to run more than ten-thousand simulations: a nominal simulation run for each technique with the remainder utilized to evaluate vulnerability to variations as described in Section 2.9. In Section 3.1, benchmarks of performance are established using classical methods for state and rate errors and optimum cost calculated in Section 2. Section 3.2, Section 3.3 and Section 3.4 describe real-time optimal utilization of feedback to establish online estimates of the solution of the modified boundary value problem described in Section 2. Each section respectively evaluates the three methods compared: *backslash\inverse*, *pinv inverse*, and *LU inverse*.

General lessons from the results include:

1. Classical feedback estimation methods are very effective at achieving very low estimation errors, but at higher costs utilizing the estimates in the decision criteria (guidance or control).
2. *Backslash\inverse* is relatively inferior to all other inverse methods
3. Singular switching generally improves state and rate estimation and costs;
4. *LU inverse* and *pinv inverse* methods perform alike with disparate strengths and weaknesses relative to each other.
5. Choosing the *pinv inverse* method as the chosen recommendation, Monte Carlo analysis reveals the residual sensitive to parameter variation is indistinguishable from the inherent sensitivity of the optimal solution when using the singular switching technique. Meanwhile, substantial vulnerability to parameter variation is revealed when singular switching is not used.
6. Lastly, omitting the complicating cross-products in the problem results in an order of magnitude high estimation errors and several orders of magnitude higher parameter estimation error. Therefore, cross-product motion decoupling is strongly recommended for all instantiations of state and rate estimation.

### 3.1. Benchmark Classical Methods

**Figure 6:**
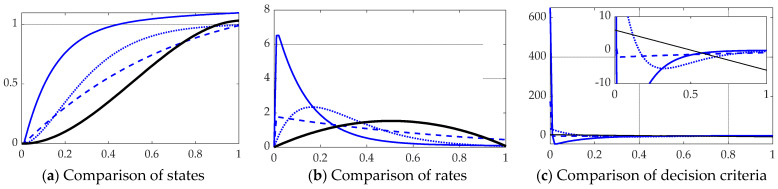
Comparison of classical methods with scaled time on the abscissae and respective ordinates titled in the subplot captions: thinner solid blue line is manually tuned PID, dashed line is linear quadratic optimal PD, dotted line is proportional plus velocity tuned to performance specification, thicker solid black line is open loop optimal per Pontryagin (Equation (31) provided for anticipated comparison). (**a**) States, (**b**) rates, and (**c**) decision criteria). Notice the solid black line representing the optimal open loop solution in subfigure (**c**) is initially positive to create movement in the desired direction, and the control switches exactly at the halfway point to a negative input, slowing progress towards the final desired end states (position and rate states). The ranges of the zoomed view in the inset are indicated by the respective scales.

Classical methods as presented in [41,42] with nonlinear decoupling loops as proposed in Equation (42) depicted in Figure 3b were implemented in SIMULINK according to Figure 4a. Computer code implementing these classical methods is presented in Appendix B, Algorithm 1. Estimation was executed by feedback of proportional plus integral plus derivative (PID), proportional plus derivative (PD), and also proportional plus velocity, and the results displayed in Figure 6, establishing the benchmark for state and rate tracking. Table 2 displays quantitative data corresponding to Figure 6’s qualitative displays. Notice the optimal estimation of state, rate, and decision criteria is also included in Figure 6 and Table 2, since the optimal cost benchmark is established by Pontryagin’s treatment in Equation (31).

### 3.2. Real-Time Optimal Methods with Backslash

**Figure 7:**
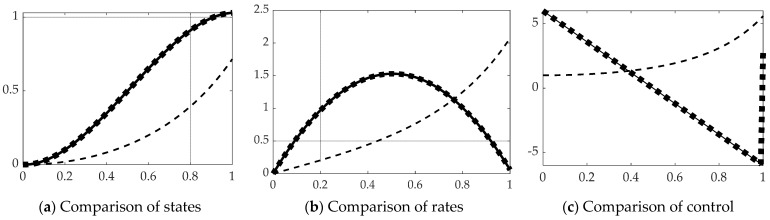
Comparison of real-time optimal methods with scaled time on the abscissae and respective ordinates titled in the subplot captions: solid line is open loop optimal (benchmark), dashed line is real-time optimal using *backslash*, thick dotted line is real-time optimal using *backslash* with singular switching. (**a**) States, (**b**) rates. Notice the solid black line representing the optimal open loop solution in subfigure (**c**) is initially positive to create movement in the desired direction, and the control switches exactly at the halfway point to a negative input, slowing progress towards the final desired end states (position and rate states). The ranges of the zoomed view in the inset are indicated by the respective scales.

This section displays the results of real-time optimal estimation using the *backslash\inverse* depicted in Figure 5b with and without singular switching displayed in Figure 4d to inverse the [*T*] in Equation (39). The results are compared to open loop optimal results per Equation (31) displayed in Figure 4c. Figure 7 reveals real-time optimal state estimation performs relatively poorly using the MATLAB *backslash\inverse*, but performance is restored to near-optimal performance when augmented with singular switching. State and rate errors are restored to essentially optimal values, while cost is restored to very near the optimal case as evidenced by the quantitative results displayed in Table 3.

**Figure 8:**
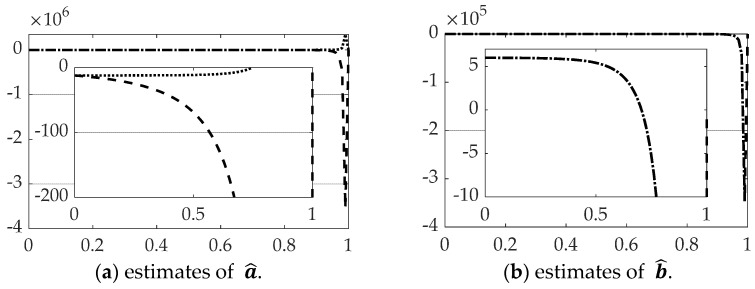
Comparison of real-time optimal methods with scaled time on the abscissae and respective ordinates titled in the subplot captions: dashed line is real-time optimal using *backslash*, dotted line is real-time optimal using *backslash* with singular switching. (**a**) Estimates of $\hat{a}$, (**b**) estimates of $\hat{b}$. The ranges of the zoomed view in the inset are indicated by the respective scales.

Figure 8 and Table 4 reveal the estimation performance of the constants of integration solving the modified two-point boundary value problem (BVP) using state and rate feedback to reset the initial conditions of the BVP. Oddly, despite relatively superior performance estimating the state and rates when using singular switching augmentation, parameter estimation is far inferior.

Section 3.1 presented the results of classical and optimal methods as benchmarks for performance. Meanwhile, Section 3.2 presented the results of implementing real-time optimal estimation with *backslash\inverse* with and without singular switching compared to the optimal benchmark. Next, Section 3.3 presents results using *pinv inverse* with and without singular switching.

### 3.3. Real-Time Optimal Methods with Pinv

**Figure 9:**
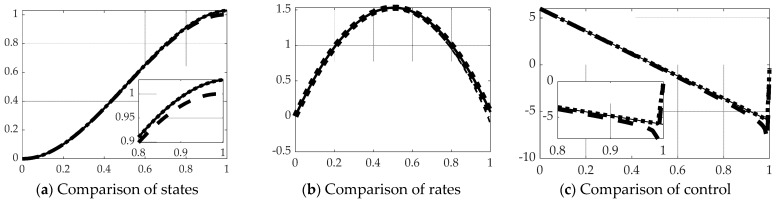
Comparison of real-time optimal methods with scaled time on the abscissae and respective ordinates titled in the subplot captions: solid line is open loop optimal (benchmark), dashed line is real-time optimal using backslash, thick dotted line is real-time optimal using *pinv* with singular switching. (**a**) States, (**b**) rates, and (**c**) decision criteria or control. The ranges of the zoomed view in the inset are indicated by the respective scales.

**Figure 10:**
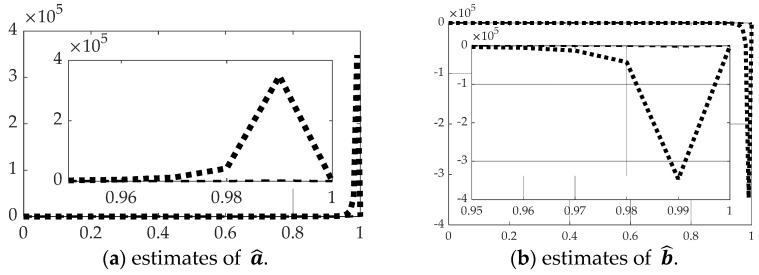
Comparison of real-time optimal methods with scaled time on the abscissae and respective ordinates titled in the subplot captions: dashed line is real-time optimal using backslash, dotted line is real-time optimal using *pinv* with singular switching. (**a**) Estimates of $\hat{a}$, (**b**) estimates of $\hat{b}$. The ranges of the zoomed view in the inset are indicated by the respective scales.

Inversion of the [*T*] matrix in Equation (39) was also accomplished by the Moore–Penrose pseudoinverse equation: ${\left[T\right]}^{-1}≅{\left[T\right]}^{†}\equiv {\left({\left[T\right]}^{T}\left[T\right]\right)}^{-1}{\left[T\right]}^{T}$. All other facets of the problem are left identical, while only the method of matrix inversion is modified resulting in state and rates estimates and comparison of control in Figure 9 with corresponding quantitative results in Table 5. Parameter estimation accuracy is displayed in Figure 10 and Table 6.

### 3.4. Real-Time Optimal Methods with Lu-Inverse

**Figure 11:**
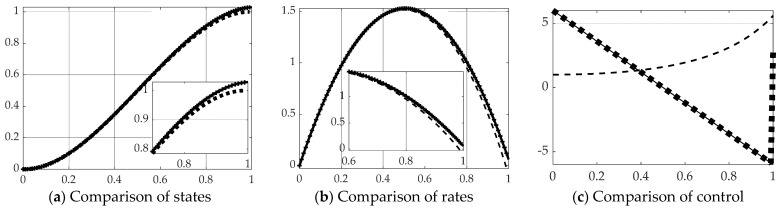
Comparison of real-time optimal methods with scaled time on the abscissae and respective ordinates titled in the subplot captions: solid line is open loop optimal (benchmark), dashed line is real-time optimal using backslash, thick dotted line is real-time optimal using *LU-inverse* with singular switching. (**a**) States, (**b**) rates, and (**c**) decision criteria or control. The ranges of the zoomed view in the inset are indicated by the respective scales.

**Figure 12:**
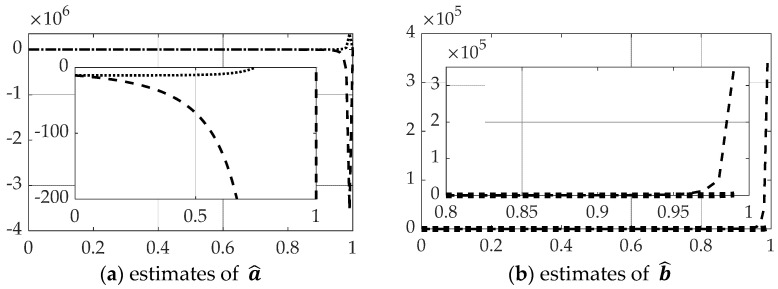
Comparison of real-time optimal methods with scaled time on the abscissae and respective ordinates titled in the subplot captions: dashed line is real-time optimal using backslash, dotted line is real-time optimal using *LU-inverse* with singular switching. (**a**) Estimates of $\hat{a}$, (**b**) estimates of $\hat{b}$. The ranges of the zoomed view in the inset are indicated by the respective scales.

Inversion of the [T] matrix in Equation (39) was also accomplished by the *LU-inverse,* which first creates a pivoted version $T_{p}$ and then inverts the product of a lower triangular matrix, [*L*] and an upper triangular matrix [*U*] based on [*T*]: ${\left[T\right]}^{-1}≅{\left[T_{p}\right]}^{-1}\equiv {\left(\left[L\right]\left[U\right]\right)}^{-1}$. All other facets of the problem are left identical, while only the method of matrix inversion is modified resulting in state and rates estimates and comparison of control in Figure 11 with corresponding quantitative results in Table 7. Parameter estimation accuracy is displayed in Figure 12 and Table 8.

### 3.5. Monte Carlo Analysis Using Pinv (with Singular Switching) and Open Loop Optimal with Cross-Product Deoupling

**Figure 13:**
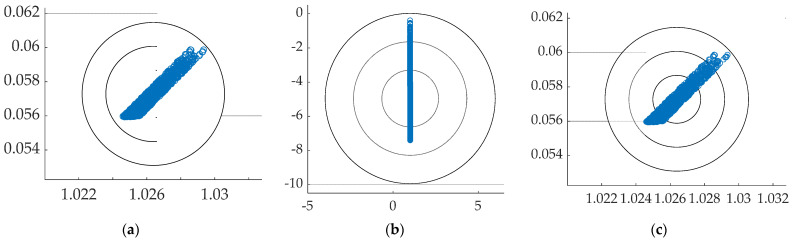
Comparison of the impacts of system variations on real-time optimal using (**a**) open loop optimal, (**b**) *pinv* without switching, (**c**) *pinv* with switching. Scaled state on the abscissae and scaled rate on the ordinates.

Over ten-thousand simulation runs were performed with 10% uniformly random variations in plant parameters (mass and mass moment of inertia). Noise was added to state and rate sensors with zero mean and standard deviation 0.01, and the results are displayed in the “scatter” plots in Figure 13 with corresponding quantitative results displayed in Table 9. Feedback implemented by resetting the initial condition of the reformulated boundary value problem (when implemented with singular switching) yielded optimal results when augmented with cross-product decoupling.

### 3.6. Comparison of Results

Section 3.1 presented the benchmark results produced by classical methods and open loop optimal mathematical solutions. Section 3.2 presented results utilizing MATLAB’s *backslash\inversion*, while Section 3.3 included results using Moore–Penrose pseudoinverse, *pinv*. Section 3.4 presented results using *LU-inverse*. Section 3.5 revealed robustness to variations in plan parameters with both state and rate sensor noise. This section consolidates the results into a single table of raw data depicted in Table 10. This data will be used to produce percent performance improvement as figures of merit in the Discussion (Section 4).

## 4. Discussion

State and rate estimation algorithms fused with noisy sensor measurements using several of the proposed methodologies achieve state-of-the art accuracies with optimality that is analytic and deterministic rather than stochastic, and therefore use very simple equations with necessarily low computational burdens. Simple relationships with small numbers of multiplications and additions are required to be comparable to the simplicity of classical methods, but optimum results are produced that exceed the modern notion of linear-quadratic optimal estimation. Implementation of non-standard feedback achieves robustness with the additional computational cost of a matrix inverse, and therefore three optional inversion methods were compared. General lessons taken with manually tuned PID as a benchmark for state and rate estimation errors, while optimal loop optimal cost is the benchmark for the cost of utilization of state estimations for guidance and control:

1. Classical feedback estimation methods (tuned per computer code is presented in Appendix B, Algorithm A1) are very effective at achieving very low estimation errors, but at higher costs utilizing the estimates in the decision criteria (guidance or control).
  - Linear-quadratic optimal estimation achieved 87% better state estimates, but over 400% poorer rate estimates compared to classical PID with costs over 2000% open-loop optimal costs.
  - Classical position plus velocity estimation achieved 90% improved state estimation with over 30% better rate estimation, but cost of implementation remains high (over 400% higher than the optimal benchmark).
2. Open loop optimal estimation established the mathematical benchmark for cost, and achieved 72% improved state estimation and 33% rate estimation errors.
3. *Backslash*\*inverse* is relatively inferior to all other inverse methods, producing 200% poorer state estimation and over 2000% poorer rate estimates with 52% reduced costs compared to the optimal benchmark;
4. Singular switching generally improves state and rate estimation and costs;
  - Singular switching with *backslash\inverse* produced 69% improvement in state estimation and 29% improvement in rate estimation with roughly optimal costs.
  - Singular switching with *LU-inverse* produced 72% improvement in state estimation and 33% improvement in rate estimation with roughly optimal costs (3% better than optimal…a numerical curiosity).
  - Singular switching with *pinv inverse* produced 69% improvement in state estimation and 29% improvement in rate estimation with roughly optimal costs (approximately identical improvement percentages to *LU-inverse* with singular switching).
5. *LU inverse* and *pinv inverse* methods perform alike with disparate strengths and weaknesses relative to each other.
6. Choosing the *pinv inverse* method as the chosen recommendation, Monte Carlo analysis reveals the residual sensitive to parameter variation is indistinguishable from the inherent sensitivity of the optimal solution when using the singular switching technique. Meanwhile, substantial vulnerability to parameter variation is revealed when singular switching is not used.
7. Lastly, omitting the complicating cross-products in the problem results in an order of magnitude higher estimation errors and several orders of magnitude higher parameter estimation error. Therefore, cross-product motion decoupling is strongly recommended for all instantiations of state and rate estimation.

### 4.1. Notes on Percentages of Performance Improvements

The choice of benchmarks for establishing percentage performance improvements leads to seemingly exaggerated numbers. The current selection of benchmarks emphasizes the strengths of the respective methods: classical feedback estimation methods are designable to achieve high accuracy but suffer from high effort by the decision criteria associated with their use. Optimal methods as instantiated here emphasize minimization of decision effort, so the benchmark for control effort is selected as optimal open loop rather than classical feedback (e.g., manually tuned PID). Percent degradation over thirty thousand percent results when compared to the optimal value (of six) as a benchmark. If the calculation had instead used the manually tuned classical PID as a benchmark, the optimal effort would exhibit an improvement over ninety-nine percent.

The final line in Table 11 illustrates the extreme penalty of not using feedback decoupling of the vector cross-products in Equation (1) representing translation due to the rotating reference. The penalty embodies the deleterious effects of neglecting treatment of the nonlinear, coupled, full six-degree-of-freedom system of equations.

### 4.2. Notes on Executability

Table 12 displays simulation run time for the eleven methods compared in the manuscript. Run-time was established by establishing the run start-time with the *tic* command, while simulation stop-time was the very first command (*toc*) following each simulation run. Simply neglecting the cross-product terms results in the fastest calculation. All the methods evaluated are of the same order of magnitude of computational burden. Simulations are depicted graphically in Figure 2, Figure 3, Figure 4 and Figure 5 and alphanumerically in Appendix B.

### 4.3. Future Research

Notice in Figure 3c sinusoidal wave input is coded using the identical time-index of the rest of the simulation. The next stages of future research will utilize this identical simulation to investigate efficacy of the proposed virtual sensoring amidst unknown wave actions. Secondly, hardware validation of key facets of this research is a logical next step.

## 5. Conclusions

Using variations of mathematical optimization to provide state, rate, and decision/control provides virtual sensing information useful as sensor replacements. In this instance, arbitrary position and rate sensors were modeled as ideal sensors, plus Gaussian random noise and algorithms were presented and compared that provide very smooth (not noisy) signals for position, rate, and acceleration (manifest in the decision/control). There was no acceleration sensor, so the notion of sensor replacement is manifest for acceleration, while the position and rate information was provided by the selected algorithm acting as a vital sensor. Real-time optimal (nonlinear) state estimation using the Moore–Penrose pseudoinverse (implemented in MATLAB using the *pinv* command) was revealed to be the most advised approach with very highly accurate estimates and essentially mathematically optimally low costs of utilization. The real-time optimal inverse calculation becomes poorly conditioned as the end-state is approached due to rank deficiency in the matrix inversion, so switching to the open loop optimal in the very end was implemented when the determinant of the matrix became nearly zero.

## Figures and Tables

**Table 1:** Software configuration for simulations reported in Section 3.

Software Version	Integration Solver	Step-Size
MATLAB R2021a	Euler (ode1)	0.01 secs

**Table 2:** Comparison of classical decision methods.

Decision Method	Final State Error	Final Rate Error	Decision Criteria/Control Effort
Classical feedback: proportional + integral + derivative (manually tuned)	0.0949	0.0850	2192
Classical feedback: proportional + derivative (linear quadratic optimal)	−0.0120	0.4341	152.7
Classical feedback: proportional + velocity (tuned to performance specs)	−0.0082	0.0555	30.82
Open loop optimal	0.0296	0.06	6

**Table 3:** Comparison of real-time optimal decision methods using *backslash* matrix inversion.

Decision Method	Final State Error	Final Rate Error	Decision Criteria/Control Effort
Open loop optimal	0.0296	0.06	6
Real-time optimal using *backslash*	0.2849	2.0762	2.863
Real-time optimal using *backslash* with singular switching	0.0296	0.06	6.0012

**Table 4:** Comparison of real-time optimal decision methods using *backslash* matrix inversion.

Decision Method	$\mathrm{Mean}\;\hat{a}$ Error	$\mathrm{Mean}\;\hat{b}$ Error
Open loop optimal	−12	6
Real-time optimal using *backslash*	21.2	−26.6
Real-time optimal using *backslash* with singular switching	4142	−4121

**Table 5:** Comparison of real-time optimal decision methods using *pinv* matrix inversion.

Decision Method	Final State Error	Final Rate Error	Decision Criteria/Control Effort
Open loop optimal	0.0296	0.060	6
Real-time optimal using *pinv*	0	−0.1088	6.6914
Real-time optimal using *pinv* with singular switching	0.0296	0.0600	6.0012
−*without* cross-product decoupling	0.3381	−0.5936	6.0012

**Table 6:** Comparison of real-time optimal decision methods using *p-inv* matrix inversion.

Decision Method	$\mathrm{Mean}\;\hat{a}$ Error	$\mathrm{Mean}\;\hat{b}$ Error
Open loop optimal	−12	6
Real-time optimal using *p-inv*	21	−26
Real-time optimal using *p-inv* with singular switching	4101	−4080

**Table 7:** Comparison of real-time optimal decision methods using *LU-inverse* matrix inversion.

Decision Method	Final State Error	Final Rate Error	Decision Criteria/Control Effort
Open loop optimal	0.0296	0.060	6
Real-time optimal using *LU-inverse*	0.0030	−0.087	6.371
Real-time optimal using *LU-inverse* with singular switching	0.0284	0.1188	5.8283

**Table 8:** Comparison of real-time optimal decision methods using *LU-inverse* matrix inversion.

Decision Method	$\mathrm{Mean}\;\hat{a}$ Error	$\mathrm{Mean}\;\hat{b}$ Error
Open loop optimal	−12	6
Real-time optimal using *LU-inverse*	21	21
Real-time optimal using *LU-inverse* with singular switching	4142	4142

**Table 9:** Impact of variations with real-time optimal decision methods using *pinv* inversion.

Decision Method	Mean Final State Error	Mean Final RateError
Open loop optimal	0.0264	0.0573
Real-time optimal using *pinv*	0.0041	−4.960
Real-time optimal using *pinv* with singular switching	0.0264	0.0573

**Table 10:** Comparison of real-time optimal decision methods.

Decision Method	Mean $\hat{a}$ Error	$\mathrm{Mean}\;\hat{b}$ Error	Mean Final Position Error	Mean Final Rate Error	Decision Criteria Cost
Classical feedback: PID (manually tuned)	--	--	0.0949	0.0850	**2192**
Classical feedback: PD (linear quadratic optimal)	--	--	−0.0120	0.4341	152.7
Classical feedback: P+V (tuned to performance specs)	--	--	−0.0082	0.0555	30.82
Open loop optimal	0	0	0.0264	**0.0573**	6.000
Real-time optimal using *Backslash (\) inverse*	9.2	−20.6	0.2849	2.0762	**2.863**
Real-time optimal using *Backslash (\)* with switching	4130	−4115	0.0296	0.0600	6.0012
Real-time optimal using *LU-inverse*	9	15	0.0041	**−4.960**	6.371
Real-time optimal using *LU-inverse* with switching	4142	4136	0.0264	0.0573	5.8283
Real-time optimal using *pinv-inverse*	21	−20	**0.0000**	−0.1088	6.6914
Real-time optimal using *pinv-inverse* with switching	4130	−4074	0.0296	0.0600	6.0012
− *without* cross-product decoupling	−47,766	47,449	**0.3381**	−0.59357	6.0012

**Table 11:** Percent improvement comparison of real-time optimal decision methods percent performance improvement.

Decision Method	Mean Final Position Error Percent Improvement	Mean Final Rate Error Percent Improvement	Decision Criteria/Control EffortPercent Compared to Optimal
Classical PID (manually tuned)	--	--	+36,433%
Classical PD (linear quadratic optimal)	87%	−411%	+2445%
Classical P+V (tuned to performance specs)	91%	35%	+414%
**Open loop optimal**	**72%**	**33%**	**--**
Real-time optimal using *Backslash (\) inverse*	−200%	−2343%	−52%
**Real-time optimal using *Backslash (\)* with switching**	**69%**	**29%**	**0%**
Real-time optimal using *LU-inverse*	96%	−5735%	+6%
Real-time optimal using *LU-inverse* with switching	**72%**	**33%**	**−3%**
Real-time optimal using *pinv-inverse*	100%	−28%	+12%
**Real-time optimal using *pinv-inverse* with switching**	**69%**	**29%**	**0%**
−*without* cross-product decoupling	−256%	−598%	0%

**Table 12:** Simulation run-time comparison.

Decision Method	Simulation Run-Time	Percent Improvement
Classical PID (manually tuned)	1.4342	- -
Classical PD (linear quadratic optimal)	1.4325	−0.1
Classical P + V (tuned to performance specs)	1.3372	−6.8
Open loop optimal	1.3511	−5.8
Real-time optimal using *Backslash (\) inverse*	1.3908	−3.0
Real-time optimal using *Backslash (\)* with switching	1.3954	−2.7
Real-time optimal using *LU-inverse*	1.3814	−3.7
Real-time optimal using *LU-inverse* with switching	1.3829	−3.6
Real-time optimal using *pinv-inverse*	1.3407	−6.5
Real-time optimal using *pinv-inverse* with switching	1.3361	−6.8
−*without* cross-product decoupling	1.3273	−7.5

## Data Availability

While the simulation codes used to produce these results are presented in the manuscript and the appendix, data supporting reported results can be obtained by contacting the corresponding author.

## Appendix A

This appendix contains a table of variable and acronym definitions used in the manuscript.

**Table A1:** Variables and acronyms.

Variable/Acronym	Definition
F, τ	external force and torque, respectively
m, I	mass and mass moment of inertia, respectively
ω,$\;\dot{\omega }$	angular velocity and acceleration, respectively
$r^{\prime }$,$\;v^{\prime },a\prime$	position and velocity, and acceleration relative to rotating reference
$\tau =I\dot{\omega }$ & F=ma′	double integrator plants
ω×Iω	cross-product translation motion due to rotating reference frame
$m\dot{\omega }\times r^{\prime }$	cross-product translation motion due to rotating reference frame
$-2m\omega \times v^{\prime }$	cross-product translation motion due to rotating reference frame
$-m\omega \times \left(\omega \times r^{\prime }\right)$	cross-product translation motion due to rotating reference frame
$E\left(x\left(t_{f}\right)\right)$	endpoint cost also known as “Mayer cost”
F(x(t),u(t))	running cost also known as “Lagrange cost”
J[x(), u()]	cost function
$e\left(x\left(t_{f}\right)\right)$	endpoint constraints
$x^{T}=\left[x,v\right]$	state vector of motion state x and rate v with initial condition $\left(x_{0},v_{0}\right)$ and final conditions $\left(x_{f-1},v_{f},t_{f-1}\right)=\left(0,0,0\right)$
u=[u]	control vector
H	Hamiltonian operator corresponding to system total energy
$\lambda^{T}$	adjoint operators, also called co-states (corresponding to each state)
$\upsilon^{T}$	endpoint costates
a,b,c,d	constants of integration
$\hat{a},\hat{b}$	estimates of constants of integration
t	time variable
$x_{f},v_{f}$	states evaluated at the final endpoint time $t_{f}$
$x_{0},v_{0}$	states evaluated at the initial time $t_{0}$
$\dot{}$	Dot notation for time-derivative $\frac{d}{dt}$
1s	notation for integration
I	mass moment of inertia, normalized to unity by scaling in Section 2.1
$y_{i}$	output at discrete time index i
i	discretization time-interval (timestep)
$\sigma_{y}^{2}$	theoretical variance of output
$\sigma_{sim}^{2}$	variance predicted in simulation of proposed methods
$\zeta ,\omega_{n}$	critical damping ratio and natural frequency
P+V	proportional plus velocity
PD,PI,PID	proportional plus derivative, integral respectively
det	determinant
RTOC	real-time optimal calculation
\	backslash matrix inversion (a MathWorks technique)
*pinv*	Moore–Penrose pseudoinverse matrix inversion
*LU*	matrix inversion by factoring and inverting a row-pivoted variant

## Appendix B

The appendix contains computer codes inserted into the InifFcn callback and StopFcn call back, respectively. Together with figures, implementation of Equations (1), (31)–(46), this is the only computer code needed to achieve the results presented in this manuscript.

**Algorithm A1.** InitFcn callbacks for SIMULINK model depicted in Figures
**Includes classical feedback tuning methods**
clear all; close all; clc; **% Initialize variables and matrices for assembly of results** timestep = 0.01; theta0 = 0; omega0 = 0; thetaD = 1; omegaD = 0; J = 1; MeanAngleNoise = 0; STDAngleNoise = 0.01; VarianceAngleNoise = (STDAngleNoise)^2/sqrt(timestep); MeanRateNoise = 0; STDRateNoise = 0.01; VarianceRateNoise=(STDRateNoise)^2/sqrt(timestep); **% PID: Rule of thumb** Kp = 1; Kd = 6.5; Ki = 5; A = [0 1; 0 0]; B = [0;1]; C = [10]; D = 0; Q = ones(2,2); R = 1*ones(1); **% PD: Ziegler-Nichols tuning for manually tuning PD** %[NUM,DEN] = ss2tf(A,B,C,D); G = tf(NUM,DEN); C = tf([Kd Kp],[1]); sisotool(G,C) **% PD: Linear Quadratic Regulator** %K = lqr(A,B,Q,R); Kp = K(1); Kd = K(2); Ki = 0; % J = 76 (ish) Kp = 1; Kd = 1.7321; Ki = 0; **% P + V: Tuning for performance specification** tr = 0.3; ts = 2; wn = 1.8/tr; zeta = 4.6/(ts*2.4); Kp = wn^2; Kv = 2*zeta*wn;

**Algorithm A2.** StopFcn callbacks for SIMULINK model depicted in Figures
% Printing scripts theta = squeeze(out.theta); omega = squeeze(out.omega); t = squeeze(out.tout); J = squeeze(out.J); u = squeeze(out.u); qxv = squeeze(out.qxv); a = squeeze(qxv(1,:)); b = squeeze(qxv(2,:)); thetaText = [‘\theta(t) J=‘, num2str(J(end)), ‘: ‘, ‘\theta(t_f)= ‘, num2str(theta(end))]; omegaText = [‘\omega(t) J=‘, num2str(J(end)), ‘: ‘, ‘\omega(t_f)= ‘, num2str(omega(end))]; ControlText = [‘\mu_u_(_t_)=‘, num2str(mean(u)) ]; figure(1); plot(t,theta, ‘:’, ‘LineWidth’, 2); hold on; plot(t,omega, ‘--’, ‘LineWidth’, 2); hold off; legend(thetaText, omegaText, ‘FontSize’,16, ‘FontName’,’Palatino Linotype’); set(gca, ‘FontSize’,16, ‘FontName’,’Palatino Linotype’);grid; figure(2); plot(t,a, ‘:’, ‘LineWidth’, 2); hold on; plot(t,b, ‘:’, ‘LineWidth’, 2); hold off; legendtexta=[‘\mu_a=‘, num2str(mean(a))]; legendtextb=[‘\mu_b=‘, num2str(mean(b))] legend(legendtexta,legendtextb,’FontSize’,16, ‘FontName’,’Palatino Linotype’); set(gca, ‘FontSize’,16, ‘FontName’,’Palatino Linotype’);grid; axis([0,1,-12.1,6.1]) figure(3); plot(t,u,’LineWidth’, 2); legend(ControlText, ‘FontSize’,16, ‘FontName’,’Palatino Linotype’); set(gca, ‘FontSize’,16, ‘FontName’,’Palatino Linotype’);grid;
